# Impact of pre- and post-meal exercise on 24-H glucose profiles in young adults who are overweight and obese

**DOI:** 10.1016/j.jesf.2025.200428

**Published:** 2025-11-17

**Authors:** Xiaoyuan Zhang, Bingqing Yang, Yilin Ho, Zhanjia Zhang, Dingfeng Wu, Junwei Qian

**Affiliations:** Department of Physical Education, Peking University, Beijing, 100871, China

**Keywords:** Exercise timing, Post-meal exercise, Continuous glucose monitoring, Obesity

## Abstract

**Objective:**

This study investigated the long-term effects of pre-meal exercise (PRE) versus post-meal exercise (POST) on glucose homeostasis in young adults who are overweight and obese using continuous glucose monitoring (CGM).

**Methods:**

In this randomized controlled trial, 34 adults (18–35 years) completed a 10-week intervention. The PRE group performed ≥30 min of moderate-intensity aerobic exercise (65 % HRmax) within 60 min before meals, whereas the POST group exercised within 0–90 min after meals, five times weekly. Participants completed two supervised sessions and three self-directed sessions weekly, monitored via heart rate belts. Habitual diets were maintained throughout the study, and physical activity was monitored using ActiGraph 3X accelerometers. Primary outcomes were 24-h CGM metrics; secondary outcomes included physical activity and metabolic indicators. Two-way ANOVA were used.

**Results:**

There is no group × time interactions reached significance for any glucose variability metric, blood biomarker, or cardiovascular parameter (all p > 0.05), confirming comparable efficacy between regimens. Significant time-effect improvements emerged in continuous glucose monitoring metrics (time in range: F = 4.85, p = 0.035) and cardiometabolic parameters (fasting glucose: F = 4.74, p = 0.038; insulin: F = 5.22, p = 0.030; triglyceride: F = 7.41, p = 0.011, systolic blood pressure: F = 4.45, p = 0.043; diastolic blood pressure: F = 15.04, p = 0.001) following the 10-week intervention. There is lower hypoglycemic exposure in the pre-meal group than the post-meal group (time below range, group effect: F = 4.54, p = 0.041), with triglycerides decreasing exclusively in pre-meal group (Δ = −.22 ± .37 mmol/L, p = 0.005). Post-meal exercise showed marginal insulin reduction (Δ = −1.92 μU/mL, p = 0.069).

**Conclusions:**

The 10-week exercise intervention significantly improved continuous glucose monitoring metrics (notably increased time in range) and cardiometabolic parameters (fasting glucose, insulin, blood pressure, triglycerides) without significant group × time interactions. The pre-meal group maintained consistently lower hypoglycemic exposure than the post-meal group. Triglyceride reduction occurred exclusively with pre-meal exercise, while insulin showed a marginal decrease only with post-meal exercise. These findings demonstrate comparable overall efficacy between timing protocols despite selective benefits for specific parameters.

## Highlights

**What is already known about this subject?**

• Exercise timing influences glycemic control, but most studies have focused on acute or short-term effects.
• Evidence suggests post-meal exercise reduces postprandial glucose levels, especially in individuals with type 2 diabetes.

**What are the new findings in your manuscript?**

• A 10-week exercise intervention demonstrating comparable 24-h glucose control (CGM-derived TIR, GV, mean glucose) between pre- and post-meal exercise in young adults who are overweight and obese.
• The pre-meal group maintained consistently lower hypoglycemic exposure than the post-meal group. Triglyceride reduction occurred exclusively with pre-meal exercise, while insulin showed a marginal decrease only with post-meal exercise.

**How might your results change the direction of research or the focus of clinical practice?**

• These findings highlight the potential for personalized exercise timing strategies based on metabolic priorities, such as lipid control or blood pressure.
• The results support the need for long-term studies using continuous glucose monitoring in non-diabetic, at-risk populations.

## 1 Introduction

The overweight and obesity rates among Chinese adults reached 50.7 %, representing a 2.5-fold increase compared with rates in 1992.^1^ The global rise in overweight and obesity rates has attracted widespread attention in the field of public health.^2^ Obesity is a well-established risk factor for hyperglycemia and diabetes.^3^ Critically, even in young adults with normoglycemia, overweight status impairs postprandial metabolism and insulin sensitivity — an early indicator of hyperglycemia risk.^4^ Alarmingly, postprandial glucose fluctuations independently amplify cardiovascular disease (CVD) risk: excursions ≥7.8 mmol/L drive a 58 % increase in CVD incidence, with risk progressively rising across normoglycemic ranges. This compels urgent intervention strategies for overweight populations preceding diabetes onset.^5^

Exercise can effectively regulate blood glucose homeostasis. Beyond established factors like exercise type, frequency, and intensity, increasing attention has been paid to exercise timing.^6^, ^7^, ^8^, ^9^, ^10^ However, research specifically addressing the interaction between exercise timing and meal status (fasted vs. fed state) remains limited.^8^ Current evidence suggests divergent metabolic outcomes dependent on this interaction: post-meal exercise (POST) may be more effective in lowering postprandial glucose levels, whereas pre-meal exercise (PRE) may enhance fat oxidation and reduce fasting glucose and insulin levels.^8^^,^^11^^,^^12^ Despite these insights, significant inconsistencies persist. Heterogeneity in study designs, including variations in exercise intensity, duration, and proximity to meals, complicates direct comparisons and consensus formation. Critically, most evidence derives from short-term acute trials,^6^ longitudinal data on sustained glycemic effects in overweight populations are scarce.^6^^,^^12^, ^13^, ^14^, ^15^ Furthermore, existing research predominantly focuses on individuals with diabetes or impaired glucose tolerance,^10^, ^11^, ^12^ while overlooking the overweight and obese populations without diabetes—a high-risk cohort exhibiting early metabolic dysregulation.^16^

Continuous glucose monitoring (CGM) has emerged as a key tool for capturing dynamic glycemic profiles, thereby offering insights into glucose homeostasis that are unattainable through fasting glucose measurements.^17^ Its utility is expanding from diabetes care to research in non-diabetic populations, including studies of metabolic interventions in overweight and obesity. CGM technology enhances self-management adherence by visualizing immediate glycemic responses to food and exercise, enabling data-driven lifestyle modifications.^18^ Our previous study demonstrated that CGM shows good agreement with reference blood glucose values in overweight or obese young adults during exercise conditions, thus supporting its reliability for daily monitoring in this population.^19^

Therefore, this study implemented a 10-week randomized trial in individuals who are overweight and obese to examine the effects of exercise timing on 24-h CGM data, as well as fasting glucose, insulin and lipid levels. Our findings provide the novel empirical evidence for optimizing exercise-meal sequencing in this high-risk population. By demonstrating whether pre-meal or post-meal exercise more effectively mitigates glucose fluctuations and improves metabolic health, we deliver actionable exercise prescriptions. These insights directly support clinical strategies to delay or prevent diabetes onset in overweight individuals through pragmatic lifestyle modification.

## 2 Methods

### 2.1 Participants

This study recruited individuals who are overweight and obese through posters online. A total of 40 individuals were screened (male: 17, female: 23). Inclusion criteria were 1) age 18–35 years; 2) body mass index (BMI) ≥ 24 kg/m^2^; 3) body fat percentage ≥19.7 % in men and ≥24.2 % in women. The exclusion criteria included 1) having serious organic lesions of the heart, liver, kidney, blood system, or chronic diseases; 2) taking medicines that affect body weight, blood glucose, or blood lipid levels; 3) being pregnant; 4) the inability to complete the physical function test or respond accurately to questionnaires; 5) the inability to follow the test procedure; and 6) refusing to sign the informed consent. According to the pre-sample size calculation, 13 participants were required per group. Considering the 20 % shedding rate, 17 participants were considered for each group, yielding a total of 34 participants across two groups. This study was approved by the Biomedical Ethics Committee of [XX] University (Approval number: IRB00001052-24062) and registered in the Chinese Clinical Trial Registry (Registration number: ChiCTR2400087763).

### 2.2 Enrollment screening

All participants were screened on-site after an initial telephone or online screening. First, they received an explanation of the study protocol and provided written informed consent. Second, participants were instructed to complete the personal information form, the physical activity readiness questionnaire (PAR-Q), the International Physical Activity Questionnaire Short Form (IPAQ-SF), and a three-day dietary record. Subsequently, baseline testing was conducted at the Body-Medicine Integration Innovation Laboratory in the second gymnasium of Peking University. After screening, three individuals were excluded: one male and one female who did not meet the inclusion criteria owing to low body fat percentage and low body weight, respectively, and one male who declined participation. Before the start of the exercise intervention, 37 individuals were randomized to PRE (n = 19) and POST groups (n = 18). Three participants withdrew from the trial during the first week owing to either discomfort with the exercise protocol or the monitoring device. Finally, 17 individuals in each group completed the intervention without any adverse effects.

### 2.3 Experiment

**Fig. 1:**
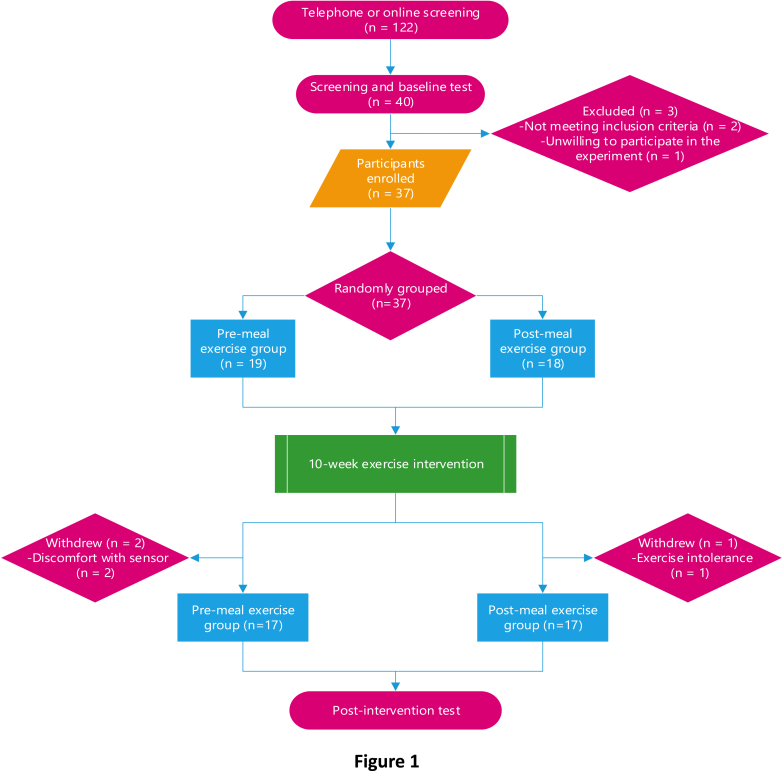
Participant flow diagram.

The participant flow diagram is seen in Fig. 1.

### 2.4 Design

This study employed random grouping to assign participants to either the PRE group or the POST group. First, the names of all participants were stored in a vector by RStudio. Then, the “sample” function was used to generate a randomly ordered list, which was divided equally into two groups to ensure randomization and minimize selection bias.

#### 2.4.1 Grouping and exercise intervention

**Fig. 2:**
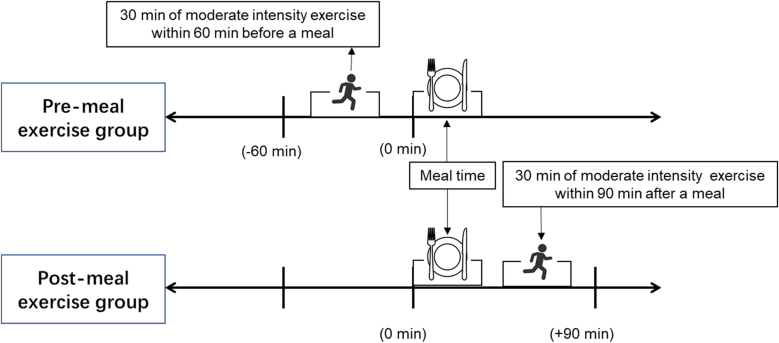
Experimental procedure flowchart.

The intervention period was 10 weeks. The PRE group exercised within 60 min before meal commencement, whereas the POST group exercised 0–90 min after starting a meal (where time 0 represents the start of the meal) (Fig. 2). The time interval between exercise and meal also affected glycemic control,^12^^,^^20^^,^^21^ therefore, all participants were asked to complete the postmeal exercise intervention within 90 min after consuming a meal to ensure exercise began before blood glucose levels peaked. Participants were instructed to select one consistent meal, breakfast or lunch, for timing interventions. Both groups exercised at the daytime between 8:00 to 14:30 to control for circadian effects. Both groups followed the same exercise regimen, differing only in timing. Given that exercise interventions for individuals who are overweight and obese aim to increase energy expenditure, reduce and control body weight, alter body composition, reduce abdominal fat, maintain or increase lean body mass, and improve cardiorespiratory and metabolic regulation, the intervention consisted of moderate-intensity aerobic exercise for at least 30 min on ≥5 days per week (including supervised sessions ≥2 times per week), performed at 65 %–75 % of HRmax (HRmax = 207–.7 × age).^22^ Exercise adherence was based on the 2 supervised weekly exercise bouts. Participants were asked to take the attendance and wear the heart rate monitoring during exercising in the gym.

The aerobic exercise was rhythmic, prolonged, and low-skill, targeting major muscle groups in the limbs and trunk. The intervention utilized digital sports technology, allowing participants to select their preferred exercise modality or equipment, such as running, swimming, rowing, or cycling. Participants wore a heart rate monitor (Yidao Health, Beijing, China) and uploaded exercise records via the Yidao Health applet. Researchers monitored the timing, duration, intensity, and volume of exercise in real time.

Participants were also required to visit the XX University Smart Health Station at least twice weekly for supervised offline exercise. Attendance and meal timing (before or after exercise) were recorded. The XX University XX Gymnasium Smart Health Station integrates traditional and intelligent exercise equipment and provides health assessments using AI through the Smart Exercise System software. This system offers participants a scientific and engaging exercise environment, with real-time feedback on intensity, energy consumption, and other metrics.

#### 2.4.2 Dietary supervision

Registered dietitians provided dietary and nutritional education based on the Dietary Guidelines for Chinese Residents (2022), covering food label reading, healthy eating strategies, and portion control. Participants were required to attend in-person lectures as well as online live streams or watch recorded videos. Regular nutritional counseling and follow-up ensured dietary balance, and participants recorded their dietary intake using the Silicon Motion app (Shenzhen, China) for three days during both the first and tenth weeks.

### 2.5 Outcomes

CGM was conducted for 14 consecutive days using the SIBIONICS CGM system to assess 24-h glucose profiles. To assess chronic glycemic adaptations, Continuous glucose monitors (CGMs) were worn for 14 consecutive days at baseline and again during the follow-up period immediately after the 10-week intervention, during which participants were instructed to avoid vigorous activity. This ensured the capture of stable glycemic states independent of acute exercise. To ensure data accuracy, the initial 24 h of CGM recordings were excluded to account for sensor stabilization, and participants were required to provide a minimum of 10 valid post-stabilization days per monitoring period; individuals failing to meet this threshold due to sensor detachment underwent monitor reapplication to complete data collection. Fasting venous blood samples were collected at baseline and post-intervention, within the time window of 48 h to 5 days after the last exercise session, to assess fasting blood glucose, insulin, and lipid profiles, including triglycerides, total cholesterol, low-density lipoprotein cholesterol (LDL-C), and high-density lipoprotein cholesterol (HDL-C) levels. Additionally, to monitor compliance and exercise levels during the intervention itself, ActiGraph 3X accelerometers were worn at baseline and during the final week of the training.

### 2.6 Statistical methods

Data were processed and analyzed using RStudio (ver. 2023.06.1 + 524, Boston, USA). Continuous variables were expressed as mean ± standard deviation (SD). Glycemic status was comprehensively assessed using a suite of metrics calculated with the EasyGV macro (https://www.phc.ox.ac.uk/research/resources/easygv). Key outcomes included the following^1^: indices of glycemic variability: including the SD, Coefficient of Variation (CV), Mean Amplitude of Glycemic Excursions (MAGE), Continuous Overall Net Glycemic Action (CONGA), Mean of Daily Differences (MODD), Maximal Glucose (MAX), Minimal Glucose (MIN), High Blood Glucose Index (HBGI), and Low Blood Glucose Index (LBGI)^2^; a summary measure: 24-h mean glucose, Glucose Management Indicator (GMI); and^3^ time-in-range profiles: Time in Range (TIR, 70–140 mg/dL), Time Above Range (TAR, >140 mg/dL), and Time Below Range (TBR, <70 mg/dL). To evaluate treatment effects over time and between groups, we conducted a two-way ANOVA with 'group' (PRE and POST) as the between-subjects factor and 'time' (pre- and post-intervention) as the within-subjects factor. Simple effects analyses with Bonferroni correction were conducted to further explore significant main effects or interactions. Given established evidence that sex influences fasting blood glucose and insulin levels,^23^^,^^24^ we included sex as a covariate for these two metabolic parameters. For all other outcome measures, standard two-way RM ANOVA was employed. A p-value <.05 was considered statistically significant.

### 2.7 Sample size calculation

Based on prior studies with similar designs, the mean postprandial glucose level in the PRE group was 11.3 mmol/L and that in the POST group was 13.7 mmol/L, with an SD of 2.2 mmol/L. The sample size of this clinical trial study is estimated considering a 10 % fallout rate and assuming a Type I error probability (α) of .05 and statistical power (1–β) of 80 %. The required sample size was calculated using the following formula:

n

$$n_{1}=n_{2}=\frac{2{\left(Z_{\alpha /2}+Z_{\beta }\right)}^{2}\times \sigma^{2}}{{\left(\mu_{2}-\mu_{1}\right)}^{2}}$$

Where

$$\mu_{1}=11.3,\mu_{2}=13.7,\sigma =2.2,\alpha =0.05,\beta =0.2$$

Substituting these values:

$$n_{1}=n_{2}=\frac{2{\left(1.96+0.84\right)}^{2}\times 2.2^{2}}{{\left(13.7-11.3\right)}^{2}}=13.17$$

Thus, 13 patients were needed in each group. Allowing for a 20 % dropout rate, 17 participants were recruited per group, resulting in a total of 34 participants across both groups.

## 3 Results

### 3.1 Baseline characteristics of the participants

A total of 34 overweight and obese participants were recruited for this study, with 17 participants in each group (PRE and POST). All participants completed the 10-week intervention. There were no significant differences between the two groups in baseline characteristics, including sex, age, height, weight, BMI, body fat percentage, and waist circumference (Table 1).

**Table 1:** Baseline characteristics of participants in each group.

	PRE group	POST group	Synthesis
n = (M, F)	17 = (6,11)	17 = (8,9)	34 = (14,20)
Age (year)	21.00 ± 2.67	21.35 ± 2.52	21.18 ± 2.56
Weight (kg)	81.18 ± 15.05	80.94 ± 11.25	81.06 ± 13.08
Body Fat (%)	34.18 ± 5.57	32.74 ± 7.04	33.46 ± 6.30
BMI (kg/m^2^)	28.24 ± 3.28	27.76 ± 2.80	28.00 ± 3.01
Waist (cm)	90.26 ± 11.98	90.23 ± 8.81	90.25 ± 10.36
GLU (mmol/L)	4.67 ± .23	4.71 ± .39	4.68 ± .31
INS (uU/mL)	11.61 ± 4.05	12.71 ± 6.05	12.16 ± 5.05
TC (mmol/L)	4.28 ± .76	4.27 ± .85	4.29 ± .78
TG (mmol/L)	1.21 ± .89	1.06 ± .64	1.13 ± .75
LDL (mmol/L)	1.38 ± .22	1.38 ± .35	1.38 ± .28
HDL (mmol/L)	2.75 ± .74	2.71 ± .70	2.73 ± .70
SBP (mmHg)	116.41 ± 14.21	119.06 ± 12.02	117.74 ± 13.03
DBP (mmHg)	74.47 ± 8.89	77.82 ± 6.83	76.15 ± 7.99
PA (MET-min/week)	1190.86 ± 771.22	1505.41 ± 823.66	1343.37 ± 800.55

Note: PRE, pre-meal exercise group; POST, post-meal exercise group; BMI, body mass index; 24-h MG, 24-h glucose mean; 24-h SD, 24-h glucose standard deviation; CV%, coefficient of variation of glucose; MAGE, mean amplitude of glucose fluctuation; CONGA, continuous overall glycemic effect; GLU, fasting glucose; INS, fasting insulin; TC, total cholesterol; TG, triglycerides; LDL, low-density lipoprotein; HDL, high-density lipoprotein; SBP, systolic blood pressure; DBP, diastolic blood pressure; PA, physical activity.

### 3.2 Comparison of 24-h glucose parameters before and after intervention

No significant Group × Time interactions emerged for 24-h mean glucose and glycemic variability markers including SD, CV, MAGE, CONGA, and MODD (all p > 0.05). Similarly, extreme glucose values (MAX, MIN) and predicted HbA1c equivalents (GMI, HbA1c) remained stable throughout the intervention period (all p > 0.05) (Table 2).

**Table 2:** Comparison of CGM metrics between groups with different exercise timing interventions.

		PRE group (n = 17)	POST group (n = 17)	Time effect (F(p), η^2^)	Group × time (F(p), η^2^)	Group effect (F(p), η^2^)
24-h mean glucose (mg/dL)	Pre-intervention	96.29 ± 4.33	96.09 ± 5.38			
	Post-intervention	98.23 ± 3.16	97.77 ± 4.22	2.701(.110), .078	.014(.907), .000	.104(.749), .003
**SD (mg/dL)**	Pre-intervention	15.95 ± 2.64	16.28 ± 2.61			
	Post-intervention	15.41 ± 1.56	16.48 ± 3.11	.214(.647), .007	.979(.330), .030	.797(.379), .024
**CV (%)**	Pre-intervention	16.59 ± 2.83	16.99 ± 2.80			
	Post-intervention	15.68 ± 1.50	16.92 ± 3.50	1.389(.247), .042	1.012(.322), .031	.932(.341), .028
**MAGE (mg/dL)**	Pre-intervention	32.90 ± 7.27	32.24 ± 4.24			
	Post-intervention	31.87 ± 4.37	33.12 ± 6.82	.006(.936), .000	1.155(.290), .035	.027(.870), .001
**CONGA (mg/dL)**	Pre-intervention	15.82 ± 3.08	15.70 ± 2.60			
	Post-intervention	15.37 ± 2.34	16.11 ± 3.21	.002(.962), .000	.921(.344), .028	.124(.728), .004
**MODD (mg/dL)**	Pre-intervention	13.55 ± 1.99	14.63 ± 1.89			
	Post-intervention	13.91 ± 1.91	14.87 ± 2.88	.635(.431), .019	.030(.862), .001	2.416(.130), .070
**MAX (mg/dL)**	Pre-intervention	172.16 ± 17.33	171.21 ± 12.04			
	Post-intervention	170.15 ± 15.38	174.60 ± 18.65	.032(.859), .001	.497(.486), .015	.195(.661), .006
**MIN (mg/dL)**	Pre-intervention	58.45 ± 11.23	53.58 ± 11.74			
	Post-intervention	59.82 ± 8.99	57.18 ± 9.41	1.122(.297), .034	.224(.639), .007	1.956(.172), .058
**GMI (%)**	Pre-intervention	5.60 ± .11	5.61 ± .13			
	Post-intervention	5.67 ± .08	5.64 ± .10	2.616(.116), .076	.570(.456), .017	.136(.714), .004
**HbA1c (%)**	Pre-intervention	4.98 ± .15	4.99 ± .18			
	Post-intervention	5.05 ± .12	5.04 ± .15	2.548(.120), .074	.102(.752), .003	.000(1.000), .000
**TIR (%)**	Pre-intervention	95.90 ± 3.23	93.98 ± 4.90			
	Post-intervention	97.33 ± 1.33	95.52 ± 3.67	4.849(.035), .132	.006(.939), .000	3.452(.072), .097
**TAR (%)**	Pre-intervention	1.92 ± 1.62	2.02 ± 1.90			
	Post-intervention	2.05 ± 1.14	2.17 ± 1.39	.215(.646), .007	.001(.978), .000	.069(.794), .002
**TBR (%)**	Pre-intervention	2.18 ± 3.36	3.99 ± 4.77			
	Post-intervention	.61 ± .58	2.31 ± 3.30	4.042(.053), .112	.006(.940), .000	4.537(.041), .124
**HBGI**	Pre-intervention	.65 ± .29	.73 ± .35			
	Post-intervention	.68 ± .24	.71 ± .41	.000(.997), .000	.163(.689), .005	.357(.554), .011
**LBGI**	Pre-intervention	2.16 ± .82	2.31 ± .97			
	Post-intervention	1.72 ± .35	2.08 ± .85	3.769(.061), .105	.350(.558), .011	1.505(.229), .045

Note: CGM, continuous glucose monitoring; PRE, pre-meal exercise group; POST, post-meal exercise group; TIR, time in range (70–140 mg/dL); TAR, time above range (>140 mg/dL); TBR, time below range (<70 mg/dL); GMI, glucose management indicator; MAGE, mean amplitude of glycemic excursions; MAX, maximal glucose; MIN, minimal glucose; CONGA, continuous overall net glycemic action; MODD, mean of daily differences; CV, coefficient of variation; HBGI, high blood glucose index; LBGI, low blood glucose index Values are mean ± SD. Statistical results from two-way repeated measures ANOVA.

Significant time effects were observed for TIR following the 10-week exercise intervention (F(1,32) = 4.85, p = 0.035, η^2^ = .132). Simple effects analyses indicated non-significant improvements within each group: PRE showed a mean increase of 1.43 % [95 % CI: −.51 %–3.37 %], p = 0.143 and POST showed a mean increase of 1.54 % [95 % CI: −.41 %–3.48 %], p = 0.117. Additionally, TBR showed a strong trend toward time-effect reduction (F(1,32) = 4.04, p = 0.053, η^2^ = .112). A significant group main effect emerged for TBR (F(1,32) = 4.54, p = 0.041, η^2^ = .124), with the PRE group demonstrating consistently lower hypoglycemic exposure. At post-intervention, PRE maintained significantly lower TBR than POST (−1.69 % [95 % CI: −.04, −3.35], p = 0.045), representing a 72 % within-group reduction from baseline (Δ = −1.57 % [95 % CI: −.77, 3.90], p = 0.181) versus 42 % reduction in POST (Δ = −1.69 % [95 % CI: −.64, 4.02], p = 0.150). The low blood glucose index (LBGI) also demonstrated a marginal decrease (F(1,32) = 3.769, p = 0.061, η^2^ = .105). These improvements occurred without significant group × time interactions (all p > 0.05), indicating comparable efficacy between pre- and post-meal exercise timing. Complete CGM metrics are presented in Table 2.

### 3.3 Comparison of fasting blood biochemical indicators and blood pressure before and after intervention

Significant time effects were observed for glucose (F(1,30) = 4.74, p = 0.038, η^2^ = .136) and insulin (F(1,30) = 5.22, p = 0.030, η^2^ = .148), though follow-up simple effects analyses revealed no statistically significant within-group changes for glucose in either the PRE (p = 0.495) or POST (p = 0.222) conditions. Insulin levels showed a marginally significant reduction within the POST group (Δ = −1.92 μU/mL, p = 0.069) but not in the PRE group (p = 0.366). No significant group × time interactions (glucose: F(1,30) = .14, p = 0.713; insulin: F(1,30) = .43, p = 0.517) or group main effects were detected, suggesting comparable glycemic outcomes between pre- and post-meal exercise regimens (Table 3).

**Table 3:** Comparison of blood biochemical indicators between groups with different exercise timing interventions.

		PRE group n = 17	POST group n = 16	Time effect *F(p), η*^*2*^	Group*time *F(p), η*^*2*^	Group effect *F(p), η*^*2*^	
GLU (mmol/L)	Pre-intervention	4.67 ± .23	4.71 ± .39				
	Post-intervention	4.63 ± .32	4.62 ± .29	4.736(.038), .136	.138(.713), .005	.014(.908), .000	
	Δ	−.04 ± .23	−.09 ± .31				
INS (uU/mL)	Pre-intervention	11.61 ± 4.05	12.71 ± 6.05				
	Post-intervention	10.78 ± 4.50	10.67 ± 4.61	5.215(.030), .148	.429(.517), .014	.029(.866), .001	
	Δ	−.83 ± 4.27	−2.04 ± 4.37				
TC (mmol/L)	Pre-intervention	4.28 ± .76	4.27 ± .85				
	Post-intervention	4.18 ± .66	4.07 ± .55	3.408(.074), .099	.356(.555), .011	.071(.791), .002	
	Δ	−.10 ± .42	−.20 ± .53				
TG (mmol/L)	Pre-intervention	1.21 ± .89	1.06 ± .64				
	Post-intervention	.99 ± .63	1.00 ± .56	7.406(.011), .193	2.586(.118), .077	.091(.765), .003	
	Δ	−.22 ± .37	−.06 ± .17				
LDL (mmol/L)	Pre-intervention	1.38 ± .22	1.38 ± .35				
	Post-intervention	1.38 ± .30	1.40 ± .32	.138(.713), .004	.042(.840), .001	.010(.921), .000	
	Δ	.01 ± .15	.02 ± .23				
HDL (mmol/L)	Pre-intervention	2.75 ± .74	2.71 ± .70				
	Post-intervention	2.71 ± .57	2.55 ± .51	2.810(.104), .083	1.118(.298), .035	.196(.661), .006	
	Δ	−.04 ± .33	−.16 ± .35				
Systolic blood pressure (mmHg)	Pre-intervention	116.41 ± 14.21	119.06 ± 12.02				
	Post-intervention	114.24 ± 9.55	115.29 ± 13.74	4.445(.043), .125	.038(.846), .001	.048(.829), .002	
	Δ	−2.18 ± 10.45	−3.76 ± 8.75				
Diastolic blood pressure (mmHg)	Pre-intervention	74.47 ± 8.89	77.82 ± 6.83				
	Post-intervention	71.82 ± 8.78	71.47 ± 7.77	15.037(.001), .327	.883(.355), .028	.321(.575), .010	
	Δ	−2.65 ± 8.83	−6.35 ± 7.66				

Note: GLU, glucose; INS, insulin; TC, total cholesterol; TG, triglycerides; LDL, low-density lipoprotein; HDL, high-density lipoprotein; Δ = Post-intervention value - Pre-intervention value. Analysis of glucose (GLU) and insulin (INS) employed two-way repeated measures ANCOVA with gender as a covariate. All other variables used standard two-way repeated measures ANOVA without covariates. Values are mean ± SD.

Diastolic blood pressure showed a robust time effect (F(1,31) = 15.04, p = 0.001, η^2^ = .327) with clinically relevant reductions in both the PRE (Δ = −6.35 ± 7.66 mmHg, p = 0.002) and POST (Δ = −2.65 ± 8.83 mmHg, p = 0.049) groups. Systolic pressure also decreased significantly over time (F(1,31) = 4.45, p = 0.043, η^2^ = .125), though within-group changes did not reach statistical significance in either condition (PRE: p = 0.108; POST: p = 0.192). Neither parameter exhibited significant group × time interactions or group effects, indicating consistent blood pressure improvements regardless of exercise timing (Table 3).

Triglycerides demonstrated a significant time effect (F(1,31) = 7.41, p = 0.011, η^2^ = .193) with simple effects confirming a statistically significant reduction specifically within the PRE (Δ = −.22 ± .37 mmol/L, p = 0.005), while the POST group (p = 0.430) showed no significant change. Total cholesterol exhibited marginal improvement over time (F(1,31) = 3.41, p = 0.074), though within-group comparisons were non-significant for both PRE (p = 0.391) and POST (p = 0.089) conditions. No significant interaction effects were observed for the lipid profiles (Table 3).

### 3.4 Exercise adherence and diet record

After 10 weeks of intervention, there was a increase in total physical activity levels in both the PRE and POST groups, a significant increase in time spent in moderate-intensity physical activity by approximately 21 min in both groups, and a decrease in sedentary time per day (94 min and 14 min, respectively). No significant change in the time spent on light- and high-intensity physical activities was observed in either group. Similarly, no significant difference was observed between the two groups (Table 4). Participants reached an average of 69.32 %–71.67 % HRmax during exercise. The attendance rate is more than 88 % (Table 5). The total energy intake in both group increased after the intervention (PRE, Δ = −103.59 ± 19.10 kcal, p < 0.01; POST, Δ = −105.94 ± 19.9 kcal, p < 0.01) (Supplemental Table 1).

**Table 4:** Description of accelerometer-derived physical activity indices in participants with different exercise timing interventions.

		PRE group n = 17	POST group n = 16
LPA (min)	Pre-intervention	63.04 ± 8.81	58.76 ± 8.33
	Post-intervention	62.28 ± 9.08	57.07 ± 9.11
MPA (min)	Pre-intervention	91.51 ± 60.6	122.08 ± 59.14
	Post-intervention	113.23 ± 69.76	143.42 ± 54.99
VPA (min)	Pre-intervention	7.84 ± 19.37	3.23 ± 6.79
	Post-intervention	6.41 ± 13.43	4.79 ± 11.41
MVPA (min)	Pre-intervention	99.35 ± 61.03	125.31 ± 55.11
	Post-intervention	119.65 ± 68.75	148.21 ± 50.59
Total PA (MET*min/week)	Pre-intervention	1190.86 ± 771.22	1454.26 ± 824.91
	Post-intervention	1551.71 ± 604.26	2030.21 ± 838.16
Average daily sitting time (min)	pre-intervention	582.35 ± 120.96	578.82 ± 113.63
	post-intervention	488.24 ± 119.96	564.71 ± 109.32

Note: PRE: Pre-meal exercise; POST: Post-meal exercise; LPA: average daily low-intensity physical activity time; MPA: average daily moderate-intensity physical activity time; VPA: average daily high-intensity physical activity time; MVPA: average daily moderate- or high-intensity physical activity time.

**Table 5:** Exercise adherence.

Parameter	PRE Group	POST Group
Attendance rate (%)	88.53 ± 7.02	90.00 ± 5.77
Mean HR	137.94 ± 10.54	142.63 ± 8.61
HR% (%HRmax)	69.32 ± 5.30	71.67 ± 4.33
Duration (min/session)	31.06 ± 1.95	30.69 ± 1.82

Note: PRE: Pre-meal exercise; POST: Post-meal exercise; HR, heart rate.

## 4 Discussion

This 10-week RCT demonstrated comparable efficacy of PRE and POST exercise in improving cardiometabolic health in overweight/obese adults. Both regimens significantly enhanced CGM-derived time-in-range, fasting glucose, insulin, triglycerides, and blood pressure (all p < 0.05). Crucially, no group × time interactions emerged for primary outcomes, indicating timing did not dictate overall metabolic benefit. However, PRE specifically reduced hypoglycemia exposure and triglycerides, while POST showed a marginal insulin-lowering trend. This suggests PRE may offer unique advantages for hypoglycemia prevention and lipid regulation in dysglycemic individuals.

Consistent evidence supports the superiority of post-meal exercise (POST) over pre-meal exercise (PRE) for attenuating acute postprandial hyperglycemia in type 2 diabetes,^10^^,^^25^^,^^26^ as well as in normoglycemic, overweight, and obese populations.^27^, ^28^, ^29^ The predominant mechanism involves enhanced muscle glucose uptake during glycemic peaks, thereby reducing glucose excursions.^26^ However, the present study showed no significant difference in glycemic control between PRE and POSTs. This divergence may reflect compensatory adaptations during sustained training, wherein preserved metabolic flexibility in our relatively healthy cohort enabled effective regulation under both fed and fasted exercise.^14^ In addition, methodological heterogeneities — including participant characteristics, intervention duration, and exercise-meal intervals — further contribute to inconsistent literature findings.^6^^,^^15^, ^16^, ^17^^,^^27^

A notable finding was the significant reduction in the hypoglycemia index and triglycerides in the PRE group. The LBGI showed a trend toward reduction (p = 0.061), which is consistent with the decrease in TBR. Therefore, the primary glycemic advantage of the PRE group appears to be its superior efficacy in significantly reducing time spent in hypoglycemia, thereby potentially offering a more favorable safety profile. Prior research has indicated that PRE may enhance 24-h fat oxidation,^6^ possibly due to glycogen depletion, especially in the liver. This increased reliance on fat as an energy source could potentially contribute to a lower risk of hypoglycemia,^16^^,^^28^ as well as lower triglycerides. Conversely, the trend toward insulin reduction with post-meal exercise may reflect acute glucose disposal effects, though this did not translate to superior 24-h glycemic control.^14^ Thus, for overweight individuals with hypoglycemia risk or hypertriglyceridemia, PRE exercise may provide targeted benefit alongside general metabolic improvement.

Although there are some differences in the absolute values compared CGM with plasma glucose, CGM effectively reflects glucose fluctuation trends.^19^^,^^25^ Current evidence on the effects of PRE versus POST on 24-h continuous glucose levels is limited. A meta-analysis^30^ of population-based intervention studies found that, while acute postprandial response followed expected physiological patterns, no significant difference in glucose levels was observed in 24-h glucose levels between the two exercise timings. Another meta-analysis^12^ comparing postprandial exercise significantly reduced both postprandial and 24-h mean glucose levels in individuals with obesity. In a few randomized controlled trials, the area under the curve of blood glucose levels during postprandial glucose tolerance tests was slightly lower in the POST group compared to that in the PRE group; however, the difference was not significant. Most existing randomized controlled trials have focused on glycemic indicators such as the oral glucose tolerance test, with few studies using CGM to assess 24-h continuous glucose levels. In the present study, examining meal-specific responses, such as the 3-h area under the curve (AUC), could offer valuable mechanistic insights into how exercise timing affects glucose control. However, the lack of precise meal timing records and the consumption of snacks and caloric beverages during the postprandial period limited our ability to analyze meal-specific responses.

Both groups in the present study demonstrated increased daily moderate-intensity physical activity and reduced sedentary time after the 10-week intervention. The use of wearable technology such as the ActiGraph 3X accelerometer highlights the potential of digital tools to promote and monitor physical activity in real-world settings. The study achieved high-quality supervision and management of the exercise intervention, ensuring good compliance and maintaining the quality of the research. Participants exercised at least twice per week under the supervision of a smart health station, which enabled accurate monitoring of heart rate and made aerobic exercise more engaging. Additionally, participants completed autonomous exercise sessions three times per week, monitored heart rate through wearable bands, and received unified management and timely reminders via the backend system. Exercise times were recorded through a digital applet, ensuring accurate execution of the intervention protocol. Adherence to the program was high, and no adverse events such as gastrointestinal discomfort were reported during POST. Dietary control is another important factor when assessing exercise effects. Although participants were instructed to maintain their original dietary habits, the total energy intake increased by 103–105 kcal after the intervention. This increase may be attributed to the change of seasons from summer to winter during the intervention period, which could have led to an increased propensity to consume more food. Alternatively, the increase in energy intake might also be due to the increased physical activity levels, which could have stimulated appetite and resulted in higher food consumption. Future studies should use digital tools to standardize and monitor diets more precisely to enhance result accuracy.

This study was a long-term randomized intervention trial, addressing the current lack of long-term studies and enhancing result reliability through supervised exercise interventions. However, some limitations remain. First, the absence of a non-exercise control group limits our ability to isolate timing-specific effects from general exercise benefits. Without a non-exercise control group, observed within-group changes may reflect external factors rather than intervention effects. Second, the relatively small sample size may limit the generalizability of the results. Third, the 10-week intervention period may not have been sufficient to capture long-term glycemic changes. In addition, self-reported dietary data may have introduced bias. Future studies should include control group, larger sample sizes, extend intervention durations, adopt more objective dietary measurements, and incorporate molecular biology and genetics to explore the mechanisms underlying exercise timing and glycemic control in greater depth.

## 5 Conclusion

The 10-week exercise intervention significantly improved fasting blood glucose, fasting insulin, resting blood pressure, and triglyceride levels, irrespective of meal-exercise timing. Critically, no group × time interactions emerged for any parameter, demonstrating comparable efficacy between pre- and post-meal regimens. Selective benefits were observed: pre-meal exercise exclusively reduced triglycerides and persistently mitigated hypoglycemic exposure, while post-meal exercise showed a trend toward insulin reduction. These findings suggest that while exercise timing does not dictate overall intervention efficacy, pre-meal scheduling may offer specific advantages for lipid regulation and hypoglycemia prevention in populations with glucose dysregulation. Future studies should address limitations regarding control groups and longer-term adherence.

## Clinical trial registration

ChiCTR2400087763.

## Disclosure

The authors declare no competing interests.

## Author contributions

X.Z. and J.Q. conceived and designed the study; X.Z., B.Y., and Y.H. performed the experiments and collected the data; X.Z. and Y.H. conducted the statistical analysis; X.Z. drafted the manuscript; Z.Z. and D.W. critically revised the intellectual content. All authors read and approved the final manuscript.

## Disclosure

The authors declare no competing interests.

## Funding

This work was supported by the 10.13039/501100001809National Natural Science Foundation of China [Grant Number 32400953].
