# Supplementary material for: Impact of pre- and post-meal exercise on 24-H glucose profiles in young adults who are overweight and obese
Source: J Exerc Sci Fit. 2025 Nov 17;24(1):200428. doi: 10.1016/j.jesf.2025.200428 (PMC12681541; doi:10.1016/j.jesf.2025.200428)
Supplement: Multimedia component 1 [file mmc1.docx]

Supplemental Table 1 Diet intake before and after the intervention

|  |  | **PRE group**  **n = 17** | **POST group**  **n = 16** |
| --- | --- | --- | --- |
| Total energy intake (kcal) | Pre-intervention | 1861.47 ± 225.63 | 1876.56 ± 156.83 |
|  | Post-intervention | 1965.06 ± 190.07* | 1982.50 ± 145.79* |
| CHO (%) | Pre-intervention | 48.12 ± 10.93 | 49.00 ± 8.50 |
|  | Post-intervention | 48.38 ± 7.16 | 48.69 ± 7.76 |
| PRO (%) | Pre-intervention | 21.36 ± 6.74 | 21.94 ± 5.01 |
|  | Post-intervention | 22.06 ± 5.50 | 22.38 ± 4.86 |
| FAT (%) | Pre-intervention | 31.41 ± 13.19 | 29.47 ± 9.43 |
|  | Post-intervention | 29.56 ± 9.87 | 28.94 ± 10.60 |

Note: PRE, pre-meal exercise group; POST, post-meal exercise group; CHO, carbohydrate; PRO, protein; FAT, fat.
